# Autoimmune Hemolytic Anemia and Hodgkin's Disease: An Unusual Pediatric Association

**DOI:** 10.1155/2016/4598587

**Published:** 2016-01-19

**Authors:** Maria Miguel Gomes, Tereza Oliva, Armando Pinto

**Affiliations:** ^1^Department of Pediatrics, Hospital de Braga, Sete Fontes, São Victor, 4710-243 Braga, Portugal; ^2^Department of Pediatrics, Portuguese Oncology Institute of Porto Francisco Gentil, 4200-072 Porto, Portugal

## Abstract

Autoimmune hemolytic anemia (AIHA) is a recognized complication of lymphoproliferative disorders. AIHA associated with Hodgkin's disease (HD) is uncommon especially in the pediatric population. The diagnosis of AIHA is usually associated with HD at the time of initial presentation or during the course of disease, but it could precede it by years to months. In adults the association of AIHA and HD is more frequent in advanced stages and in the nodular sclerosis and mixed cellularity type HD. Warm immune hemolytic anemia is mainly controlled with steroids and chemotherapy. We report a case of a pediatric patient with direct antiglobulin positive test at the diagnosis of a late relapse of stage III B mixed cellularity type HD.

## 1. Introduction

Autoimmune hemolytic anemia (AIHA) is an acquired clinical condition which is characterized by the production of autoantibodies that bind to the surface of circulating erythrocytes, leading to hemolysis and decreased survival of the red blood cells [1, 2]. The direct antiglobulin test (DAT) is considered to be a cornerstone in establishing the diagnosis of AIHA, since there is uptake of autoantibodies and/or complement components onto the affected red blood cells [1, 2]. In less than 5 percent of the pediatric AIHA cases, DAT is negative due to low amount of IgG on the erythrocytes [1, 2].

Hodgkin's disease (HD) classically presents with lymph node enlargement with or without B symptoms (unexplained weight loss exceeding 10% of body weight in 6 months, fever, and drenching night sweats) [2]. Anemia is a common manifestation of HD and different mechanisms which contribute to anemia include anemia of chronic disease, reduced red cell survival, bone marrow infiltration, AIHA, and bone marrow suppression by chemotherapy [2].

AIHA is a recognized complication of lymphoproliferative disorders [2, 3]. AIHA associated with HD is uncommon especially in the pediatric population [3]. The diagnosis of AIHA is usually associated with HD at the time of initial presentation or during the course of disease, but it could precede it by years to months [3, 4].

In adults the association of AIHA and HD is more frequent in advanced stages (III and IV) and in the nodular sclerosis and mixed cellularity type HD [2, 3].

We report here a case of DAT positive AIHA at the diagnosis of a late relapse of stage III B mixed cellularity type HD.

## 2. Case Description

A 4-year-old female child presented with bilateral cervical lymphadenopathy with six-month duration associated with intermittent fever and night sweats. She was previously healthy and there was no family history of oncologic or immunologic diseases. On examination she had a left supraclavicular lymph node of 3 cm (long axis) and a left cluster of lymph nodes of 6 cm (long axis). Both were nontender and firm. Spleen was palpable, firm, and nontender below the costal margin. The rest of the examination was unremarkable. Complete blood cell count was normal: hemoglobin concentration 12.0 g/dL, hematocrit 39%, mean corpuscular volume 81 fL, mean corpuscular hemoglobin 32.1 pg, white blood cell count 4.9 × 10^9^/L, and platelet count 309 × 10^9^/L. Sedimentation rate was elevated (66/106 mm on first and second hour). Immunoglobulins were in the normal range and viral serologies were negative for Cytomegalovirus, herpes simplex I and herpes simplex II, varicella-zoster, Hepatitis A, B, and C Virus, Human Immunodeficiency Virus, and Epstein Barr Virus. Computed tomography (CT) scans revealed multiple cervical, supraclavicular, mediastinal, lumbar-aortic, mesenteric, and hepatic hilar lymph node enlargement, of maximum 3 cm (long axis) and homogeneous splenomegaly (10 cm long axis). Scintigraphy with gallium-67 citrate showed diffused fixation on cervical and left supraclavicular lymph nodes. Bone marrow aspiration and bone marrow biopsy were normal. Biopsy of cervical lymph node showed immunophenotype positive for CD30 and CD15, and negative for CD45, CD20, CD2, CD3, epithelial membrane antigen (EMA), and anaplastic lymphoma kinase (ALK). Histological examination confirmed the diagnosis of stage III B of mixed cellularity type HD. According to the German Society of Pediatric Oncology and Hematology Hodgkin Lymphoma Trial 95 (GPOH-HD 95), the patient received two intensive cycles of chemotherapy with vincristine 1.5 mg/m^2^/day, procarbazine 100 mg/m^2^/day, prednisone 60 mg/m^2^/day, and Adriamycin 40 mg/m^2^/day (OPPA) and two cycles of cyclophosphamide 600 mg/m^2^/day, vincristine 1.4 mg/m^2^/day, procarbazine 100 mg/m^2^/day, and prednisone 60 mg/m^2^/day (COPP). A partial remission (PR) was obtained and the treatment proceeded with 16 Gy mantle radiotherapy (from the upper cervical region till the mediastinum). In the posttreatment evaluation, the CT scans were normal and the 18-Fluorodeoxyglucose Positron Emission Tomography (PET) scan was negative.

She was evaluated in periodic follow-up consultations and remained asymptomatic with no hematological or imaging alterations.

Seven years later, the 11-year-old child presented with microcytic and hypochromic anemia: hemoglobin concentration 9.4 g/dL, hematocrit 33.5%, mean corpuscular volume 73.5 fL, mean corpuscular hemoglobin 21.1 pg, white blood cell count 7.5 × 10^9^/L, and platelet count 279 × 10^9^/L. She did not respond to the treatment with iron hydroxide 6 mg/kg/day (Table 1). One month later she presented with fever and abdominal pain and still had microcytic and hypochromic anemia (Table 1). On examination she had a nontender mass on the umbilical region of 4 cm (long axis) and hepatosplenomegaly. The rest of the examination was unremarkable. The CT scans revealed a cluster of lumbar-aortic lymph nodes of 5.6 × 4 cm between the superior mesenteric artery and the bifurcation of the iliac arteries and mild hepatosplenomegaly. The PET scan showed intense fixation of the abdominal mass and lumbar-aortic lymph nodes. Bone marrow aspiration was normocellular. Bone marrow biopsy revealed erythroid hyperplasia. Laboratory investigations showed normal lactate dehydrogenase, liver function, total and indirect bilirubin, alanine and aspartate transaminase, and immunoglobulins. Viral serology was negative. Peripheral blood smear showed markedly anisocytosis, polychromasia, and spherocytosis. The DAT was positive for IgG and C3d and a diagnosis of AIHA was made. Biopsy of the abdominal mass confirmed late relapse of the previous diagnosed mixed cellularity type HD.

According to the relapse treatment in EuroNet Pediatric Hodgkin's Disease Group, the patient completed two cycles of chemotherapy with ifosfamide 2000 mg/m^2^/day, etoposide 125 mg/m^2^/day, and prednisolone 100 mg/m^2^/day (IEP) and also two cycles with Adriamycin 25 mg/m^2^/day, bleomycin 10 mg/m^2^/day, vinblastine 6 mg/m^2^/day, and dacarbazine 375 mg/m^2^/day (ABVD). The treatment proceeded with 28.8 Gy lumbar-aortic and splenic hilum radiotherapy. A complete remission was obtained in the posttreatment evaluation. The CT scans showed a hypodense nodular lesion of 2 cm near the left renal hilum and the PET scan was negative. After the first cycle, the hemoglobin level returned to normal values (Table 1). Prednisolone reduction was attempted after 4 weeks and slow tapering was continued for three months.

The patient was evaluated in periodic follow-up consultations. In the last follow-up, being 14 years old, she remained with sustained complete remission: asymptomatic with no other autoimmune conditions and no hematological or imaging alterations.

## 3. Discussion

The association between HD and DAT positive AIHA in adults has ranged from 0.2% in one European study [4] to 3-4% in two American studies [3, 5]. It is unusual in the pediatric population, but there are no specific rates [6–8]. AIHA may occur in lymphoproliferative diseases, especially chronic lymphocytic leukemia (5–10%) [9–11] and non-Hodgkin's lymphoma (2-3%) [12], but is rarely seen in HD [13, 14]. Sporadic case reports and reviews have shown that when AIHA occurs in HD, it happens mostly in stages III and IV of nodular sclerosis or mixed cellularity type HD [15].

The AIHA is usually detected at the time of diagnosis or in a relapse [5]. The exact mechanism of AIHA in HD is complex and unclear. It is a possibility that the autoantibodies are directly produced by tumor cells or are related to an immune regulatory phenomenon. Possibly there is an autoimmune process at the early stages of HD in which antibodies are produced against the tumor and red blood cells as a paraneoplastic phenomenon. The antibodies initially prevent tumor's growth but when it escapes the antitumor effect it manifests as HD. Also, patients with HD are known to have an impaired cell-mediated immune response due to decreased number and defective T-lymphocytes function. Decreased number of cytotoxic T-cells could lead to excessive autoantibody production partly due to hyperactivation of B-cells.

Corticosteroids are the mainstay of therapy for AIHA. The initial dose is 1–1.5 mg/kg. Response may only be evident in 7 days. Dose reduction has to be attempted after 4 weeks and slow tapered to be continued for three months. Our patient had a rapid and sustained response to oral steroid therapy. Her clinical course improved without further need for red blood cell transfusions.

DAT was repeated 6 months after the treatment and became negative.

This case shows that we should be aware of the possibility of AIHA in patients with HD presenting with anemia.

## Figures and Tables

**Table 1:** Hematologic values.

Parameters	Values at presentation	Values 1 month later	Values after 1 cycle of treatment	Reference
Hemoglobin concentration	9.4	8.0	12.3	Lower limit of normal, 12.0 g/dL

Hematocrit	33.5	26	38.2	Lower limit of normal, 36%

Mean corpuscular volume	73.5	75	80.7	Lower limit of normal, 78 fL

Mean corpuscular hemoglobin	21.1	24	28	Lower limit of normal, 28 pg

Reticulocytes	10%	9%	1	0.5–2.5%

Iron	24	26	30	23–123 *μ*mol/L

Total iron-binding capacity	39.1	40.5	55	54–68 *μ*mol/L

Ferritin	254	463	150	12–150 *μ*g/L

Leukocytes	10.3 × 10^3^	9.9 × 10^3^	4.0 × 10^3^	5.0–14.5 × 10^3^/*µ*L
Neutrophils	6.9 × 10^3^	5.4 × 10^3^	1.5 × 10^3^	1.0–8.5 × 10^3^/*µ*L
Lymphocytes	1.8 × 10^3^	2.3 × 10^3^	2.0 × 10^3^	1.5–8.0 × 10^3^/*µ*L

Platelets	341 × 10^3^	239 × 10^3^	200 × 10^3^	200–450 × 10^3^/*µ*L

## Abbreviations

AIHA: Autoimmune hemolytic anemia
CT: Computed tomography
DAT: Direct antiglobulin test
HD: Hodgkin's disease
PET: Positron Emission Tomography.
